# Integrated metabolomic and transcriptomic analysis provides insights into the browning of walnut endocarps

**DOI:** 10.3389/fpls.2025.1582209

**Published:** 2025-05-09

**Authors:** Yifeng Wang, Mingxia Wang, Yaonian Chen, Wenbin Hu, Shuling Zhao

**Affiliations:** ^1^ College of A & F Technology, Longnan Normal University, Longnan, Gansu, China; ^2^ Chengxian Walnut Technology Service Center, Longnan, Gansu, China

**Keywords:** walnut, endocarps, browning, metabolomics, transcriptomics, RNA-seq

## Abstract

Walnut (*Juglans regia L.*) is an important woody plant worldwide, and endocarp color affects the economic value of walnut. During the postharvest processing and storage of walnut, the endocarp often undergoes browning. Browning has become a major obstacle to walnut storage, not only affecting the taste and flavor of walnuts but also reducing their nutritional quality and commercial value. In the present study, to elucidate the molecular mechanism of walnut endocarp browning, analyses of the ultrastructure, physiological characteristics, and transcriptomic and metabolomic data of walnut endocarps at different storage periods were performed. Integrated transcriptomic and metabolomic analysis showed that many differentially expressed genes (DEGs) and metabolites (DAMs) were involved in the pathways of flavonoid biosynthesis, amino acid biosynthesis, unsaturated fatty acid biosynthesis, phenylalanine metabolism, and oxidative phosphorylation. Among them, the expression levels of DEGs related to flavonoid metabolism and antioxidant activity had significant differences during their storage periods. In addition, the expression of stress-related transcription factors AP2/ERF, WRKY, bHLH, HSF, and MYB involved in the phenylpropanoid metabolic pathway was significantly upregulated during the browning process. This study comprehensively analyzed the causes of walnut endocarp browning, providing insights for studying the molecular mechanism of endocarp browning during storage and processing of walnuts and other fruits.

## Introduction

Walnut (*Juglans regia L.*) is an important nutritious tree with high economic value and is widely planted by producers around the world (Jin et al., 2022). The nucleolus contains a lot of proteins, unsaturated fatty acids, minerals, and vitamins, especially rich in essential unsaturated fatty acids ω-3, ω-6, etc., so it is beneficial for human growth and development and disease prevention (Djuricic and Calder, 2021). However, the nucleolus is prone to oxidation and rancidity due to its difficulty to store owing to its high unsaturated fatty acid content. As a thin film wrapped outside the nucleolus, the endocarp is rich in polyphenols such as tannins, which can scavenge free radicals and block the extension of oxidation reaction, thus protecting the nutritional components of the nucleolus. Meanwhile, endocarps rich in polyphenols can be used as an important source of natural antioxidants in the food processing industry (Yang et al., 2009). In addition, endocarp color is the most intuitive quality index for evaluating walnut products. The bright color and complete structure of the endocarp indicates that the nucleolus is fresh and nutrient loss is less (Wu et al., 2024). However, the inner seed coat of a walnut kernel is prone to browning during postharvest drying or storage and loses its protective effect on the kernel, accelerating oil rancidity and protein oxidative damage. Therefore, browning not only affects the taste and flavor of walnuts but also reduces their nutritional quality and commodity value, which has become a major obstacle to walnut storage.

Postharvest browning is a physiological disorder of fruit, which is caused by many reasons, such as aging, mechanical damage, environmental stress, and energy shortage (Zhang et al., 2023). It is well known that browning of fruit is divided into enzymatic browning and non-enzymatic browning; the former is considered to be the main cause (Lin et al., 2024). Phenols are mainly oxidized to brown ketones through the shikimic acid pathway and the phenylalanine pathway, followed by a series of dehydration, polymerization, and finally the formation of dark quinones (Martinez and Whitaker, 1995). The theory of regional distribution of substrate phenols and oxidases is one of the most recognized mechanisms of enzymatic browning. When the cell compartmentalization is lost, the substrate phenols will bind to the enzyme to form black substances, which will cause browning (Queiroz et al., 2011). Walnut’s endocarp is prone to different degrees of browning due to its rich polyphenols. The color after browning depends on the content of polyphenols and the color of metabolites. For example, chlorogenic acid is dark orange, catechin is bright yellow, and juglone is darker (Luo et al., 2016). At present, most of the studies on the browning mechanism of agricultural products and the inhibition of browning focus on the preservation of fresh fruits and vegetables. There are few reports on the mechanism of browning of the inner seed coat during the storage of walnut nuts. Based on this, the walnut variety ‘Qingxiang’ was used as the experimental material in this study. The ultrastructure observation, physiological index determination, and comprehensive analysis of transcriptomics and metabolomics were carried out during storage to reveal the physiological mechanism of browning during storage. It has important theoretical significance for the study of walnut browning regulation and the development of post-harvest treatment and high-quality storage technology.

## Materials and methods

### Plant material and tissue collection

Walnuts *Juglans regia* L. cv. Qingxiang with green husk were harvested from a demonstration garden (Chengxian, GPRS: Lo-105.76812; La-33.74787) in Longnan, Gansu Province, China. After harvest, the walnuts were divided into seven groups (each group had three biological replicates with 30 fruits per replicate) after being peeled off. The first group of walnuts was taken out on the day of sampling as the material for 0 days, and the remaining walnuts underwent accelerated storage in an artificial climate box (temperature 25°C, light cycle 16 h during the day, 8 h at night). The samples were taken every 15 days until the 90th day. After each sampling, the shell was peeled off and the nucleolus was taken out. Except for the samples used for appearance and cell ultrastructure analysis, the remaining nucleolus was soaked in distilled water for 40 min to separate the endocarps. The separated endocarp was ground into powder with liquid nitrogen and placed in a refrigerator at −80°C for subsequent analysis.

### Endocarp color andcell ultrastructure observation and browning degree determination

The half of the whole seed kernel was taken and photographed with a Canon IXUS 240 camera to record the color change of the nucleolus seed coat during each storage period. Ultrathin sections (50 nm thick) were embedded in epoxy resin and stained with 2% uranyl acetate-saturated alcohol solution for 8 min in the dark. The seed coat cells were washed three times with 70% alcohol and three times with ultrapure water, stained with 2.6% lead citrate solution for 8 min, washed three times with ultrapure water, dried with filter paper, and observed and photographed under a transmission electron microscope, and the ultrastructure of the seed coat cells was observed in different storage periods. The browning degree was determined according to the method of Chen et al. (2024). The powder of the inner seed was weighed at 2 g, 10 mL of 95% ethanol was added, 4,000×g was centrifuged for 20 min, and the supernatant was taken. The absorbance value was measured at 420 nm, and the browning degree was expressed as A420×10.

### Relative electrical conductivity (REC)

According to the method described by Ma et al. (2021), with minor modifications, the walnut endocarp powder (2 g) was mixed with 20 mL of double-distilled water and incubated at 25°C for 1 h. The electrical conductivity (P1) was determined by using a conductometer (DDS-307A, INESA Instrument Co., Ltd., Shanghai, China). Then, the mixed walnut solution was boiled in a water bath for 15 min. Then, it was cooled to 20°C and balanced at 20°C for 20 min. After shaking, the value of the electrical conductivity (P2) was recorded. The electrical conductivity of the walnut samples was calculated using the following equation: (Equation 1)


(1) $$\text{REC }\left(100\%\right)=\frac{{\text{P}}_{1}}{{\text{P}}_{2}}\times 100\%$$

### Changes of MDA contents in walnut endocarps

The MDA contents was determined by the thiobarbituric acid (TBA) method, according to the method described by Gao et al. (2017), with slight changes. The walnut endocarps (1 g) were ground with 2 mL of 10% trichloroacetic acid (TCA), and then 8 mL TCA was added for further grinding. After centrifuging at 3,000× g for 10 min, the supernatant was collected for the analyses of MDA contents. The supernatant (4 mL) was added to a 10-mL test tube (2 mL of distilled water was added to the control), and then 4 mL of 0.6% TBA was added to mix. The mixture was placed in a boiling water bath for 15 min and then quickly placed in an ice water bath. After cooling, the mixture was centrifuged at 3,000×g for 10 min. The absorbances of the supernatant were measured at 532, 600, and 450 nm. The calculation formula of MDA content is as follows (2) (Equation 2):


(2) $$\text{MDA }\left(\text{µmol}\cdot {\text{g}}^{-1\;}\text{FW}\right)=\frac{\left[6.45\times \left({\text{A}}_{532}-{\text{A}}_{600}\right)-0.56\times {\text{A}}_{450}\right]\times {\text{V}}_{1}\times \text{V}}{\text{W}\times \text{V}2\times 1000}$$

In formula (2), A is the absorbance at the corresponding wavelength, V1 is the total volume of the reaction solution (mL), V is the total volume of the extract (mL), V_2_ is the volume of the extract in the reaction solution (mL), and W is the weight of walnut endocarps (g).

### Total phenol analysis

According to the method described by Ma et al. (2021), with minor modifications, the 0.250-g walnut endocarp powder sample was weighed in a 10-mL centrifuge tube, added with 5 mL of 80% ethanol (v/v), ultrasonically extracted at 60°C for 30 min, and centrifuged at 8,000 × g for 10 min, and the supernatant was collected as the total phenol extract. The total phenol content of the walnut endocarps was determined using the Folin–Ciocalteu assay. Specifically, 0.1 mL of total phenol extract was placed in a 10-mL centrifuge tube, and 2.5 mL of 10% Folin reagent was added to shake it up and stand at room temperature for 2 min; then, 2 mL of 7.5% Na_2_CO_3_ solution was added to fully mix and react in the dark for 1 h. Afterward, the absorbance changes were recorded at 760 nm using a spectrophotometer (UV-3100, Mapada Co., Ltd., Shanghai, China). Gallic acid was used to obtain the standard curve.

### Enzymatic activities

About 0.1 g of walnut endocarps was weighed and homogenized in 1 mL phosphate buffer. The supernatants were centrifuged at 10,000 × g for 20 min at 4°C. The supernatants were kept at 4°C prior to polyphenol oxidase (PPO), superoxide dismutase (SOD), peroxidase (POD), catalase (CAT), lipoxygenase (LOX), and Na^+^, K^+^-ATP activity determination.

#### PPO activities

0.7 mL of phosphate buffer and 0.2 mL of catechol were mixed in a centrifuge tube and incubated at 25°C for 10 min, and then 0.1 mL of the test solution was added (0.1 mL of the test solution after boiling for 5 min was added to the control). After mixing, the reaction was carried out at 25°C for 10 min and then immediately in a boiling water bath for 5 min. After centrifugation at 5,000 rpm for 10 min, the absorbance value was measured at 410 nm (Fang et al., 2015). The absorbance at 410 nm was changed by 0.01 as an enzyme activity unit (U g^−1^ min^−1^) per g tissue per min in the per mL system.

### Activities of antioxidant enzymes: SOD, CAT, and POD

The activity of SOD was obtained using 1.5 mL of working solution containing 0.9 mL enzyme extract, 0.15 mL of methionine, 0.15 mL of NBT, and 0.15 mL of riboflavin. One unit of SOD activity is defined as the amount of enzyme that causes 50% inhibition of nitroblue tetrazolium.

The CAT activity of the walnut endocarps was measured by mixing 0.2 mL of enzyme extract, 1.6 mL of the phosphate buffer (0.1 M, pH 6.8), and 0.2 mL of H_2_O_2_. One unit of CAT activity was defined as an increase of 0.01 absorbance unit per min at 240 nm.

The absorbance changes of a mixed solution containing 0.2 mL of enzyme extract, 0.58 mL of the phosphate buffer (0.1 M, pH 6.8), 0.2 mL of H_2_O_2_ (2%), and 0.2 mL of guaiacol (25 mM) was recorded at 410 nm for 5 min to analyze the POD activities of the walnut kernels. One unit of POD activity was defined as an increase of 0.01 absorbance units per min.

#### LOX and Na^+^, K^+^-ATPase activity

The kit method (the kit was purchased from Solarbio Technology Co., Ltd., Beijing, China) was completed according to the operation instructions of the corresponding kit. For LOX activity (kit number: YA0602), the catalytic absorbance value of each gram of sample per minute at 25°C was changed by 0.001 units as an enzyme activity unit (U·g^−1^·min^−1^) under the 1 mL system. For Na^+^, K^+^-ATPase activity (kit number: YA0602), the Na^+^, K^+^-ATPase activity unit (U g^−1^ h^−1^) was defined as the amount of 1 µmoL inorganic phosphorus produced by Na^+^, K^+^-ATPase to decompose ATP per gram of tissue per hour.

### Transcriptome analysis

The RNA extraction and sequencing were performed by Matville Biotechnology Co., Ltd. (Wuhan, China). Total RNA was extracted by ethanol precipitation and CTAB-pBIOZOL. The purity of RNA was detected by a nanophotometer spectrophotometer, the concentration of RNA was detected by a Qubit 4.0 fluorescent/MD microplate reader, and the integrity of RNA was detected by a Qsep400 biological analyzer. Using the structural feature that most mRNAs in eukaryotes have polyA tails, mRNAs with polyA tails were enriched by Oligo (dT) magnetic beads, and RNA libraries were constructed by random hexamer primer reverse transcription. The Illumina platform was used for sequencing, and the off-line data (raw data) was tested for sequence quality by FastQC v 0.11.9; Trimmomatic 0.39 was used to filter the low-quality bases and adaptor sequences in raw data to obtain clean data. The obtained sequence was aligned to the walnut genome (Chandler v 2.0 https://www.ncbi.nlm.nih.gov/assembly/GCF_001411555.2) to obtain mapped data, and the expression level (TPM) of all genes in each sample was calculated by RSEM. With |log2FC| ≥1 and P<0.05 as the standard, differentially expressed genes (DEGs) were screened, and then GO enrichment and KEGG enrichment analyses were performed.

### Widely targeted metabolomics analysis

The walnut endocarp samples at three different storage periods of 0, 15, and 30 days were selected for metabolomics analysis using a widely targeted metabolomics technique. The extraction and analysis of metabolites were completed by Metware Biotechnology Co., Ltd. (Wuhan, China). The sample powder (50 mg) was extracted with 1,200 µL of 70% methanol, and water internal standard extract precooled at −20°C in advance was added. The extract was vortexed once every 30 min six times, the supernatant was taken after centrifugation at 12,000 rpm for 3 min, and the sample was filtered with a 0.22-µm microporous membrane and stored in an injection bottle. Ultra-performance liquid chromatography and tandem mass spectrometry (MS/MS) were used for data collection. Metabolites were identified based on the MWDB (Metware database).

## Results

### The changes of cell ultrastructure and browning degree of walnut endocarps

**Figure 1:**
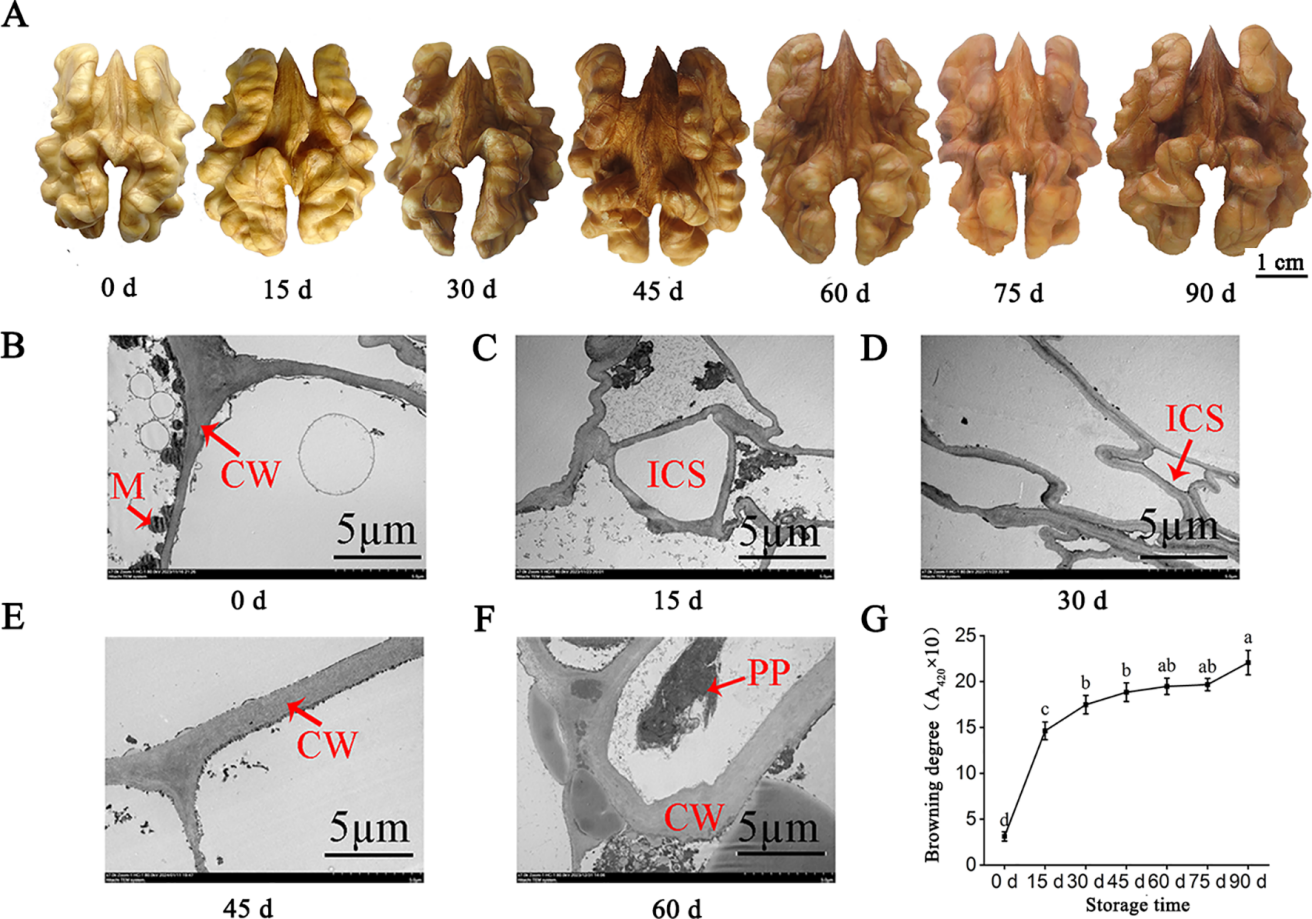
Apparent changes of walnut endocarps during storage. **(A)** Browning phenotypes of walnut endocarps during fruit storage. **(B-F)** Ultrastructural changes of walnut endocarps. **(G)** Browning degree of walnut endocarps. CW, cell wall; M, mitochondria; ICS, intercellular space; PP, polyphenol.

The endocarp color of the walnut reflects its degree of browning. In this study, the endocarp color deepened with the extension of storage times. The endocarp color was light yellow at the initial stage of 0 days, turned yellow at 15 days, and turned light brown at 30 days (**Figure 1A**). These results indicated that the endocarp browning was more serious in the early stage of storage. At 0 days, the cell structure of walnut endocarps was relatively complete, and the cell wall boundary was clear with a high level of electron density and mitochondrial content. At 15 days of storage, the cell structure began to change, the vacuole had inclusion exudation, the mitochondria began to disintegrate far away from the cell wall, and the electron density of the cell wall decreased. After 30 days, the cell wall began to thicken and the inclusions were severely degraded. After 60 days of storage, the cell wall began to crack (**Figures 1B–F**). The results also showed that with the prolongation of storage times, the browning degree of endocarps increased, and the browning degree of 0-15 days increased the most (from 3.12 to 14.63) (P< 0.05). The growth rate of the browning degree in 30-75 days was small, and the difference was not significant (P > 0.05). The browning degree was the highest (22.03) at 90 days of storage, which was 18.91 higher than that at 0 days (**Figure 1G**).

### The changes of physiological and biochemical indexes of walnut endocarps during storage

**Figure 2:**
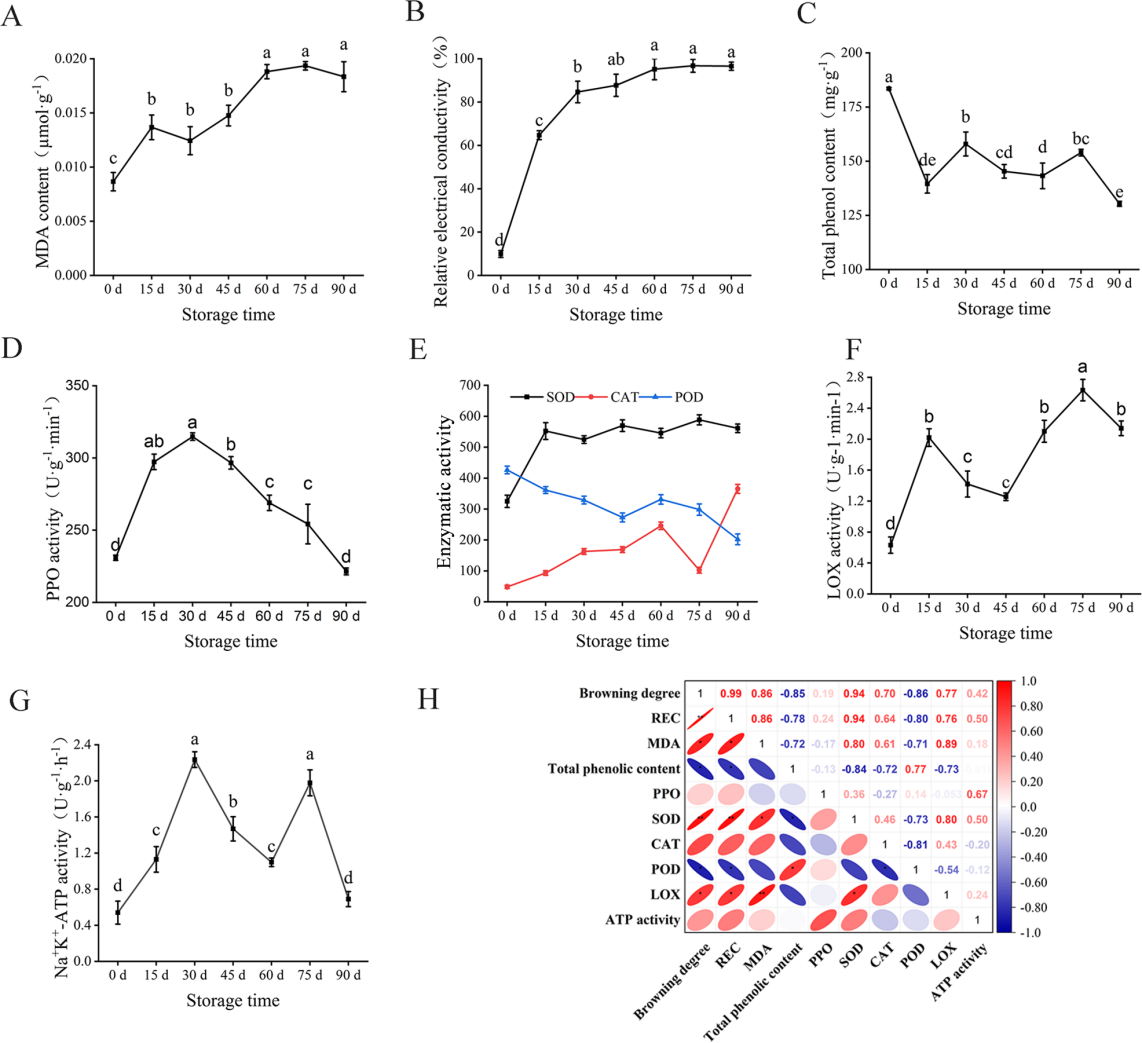
Changes of physiological and biochemical indexes of walnut endocarps during storage. **(A)** MDA content. **(B)** Relative electrical conductivity. **(C)** Tal phenolic content. **(D)** PPO activity. **(E)** SOD, CAT, and POD activity. **(F)** LOX activity. **(G)** Na^+^, K^+^-ATP activity. **(H)** Correlation analysis of browning index of walnut endocarps. * marked in the ellipse showing the significant differences in the pairwise comparison evaluated by Student *t*-test.

MDA is the product of lipid peroxidation of the plant cell membrane, which can interact with nucleic acid or amino acid residues to reduce the stability of the cell membrane and lead to electrolyte leakage (Babalar et al., 2018; Mittler et al., 2004). In this study, the MDA content of walnut endocarps showed an overall upward trend with the rise of storage time (**Figure 2A**). The largest upward trend in 0~15 days (from 0.0086 to 0.0147 µmol·g^−1^) and a downturn were observed when stored at 15~30 days and then increased rapidly. After 60 days, the changes of MDA content tended to slow. At the end of storage (90 days), the MDA content was 21-fold higher than at the beginning of storage (0 days). The relative conductivity (REC) increased during the whole storage period, and the fastest growth rate (from 9.89% to 64.71%) was observed at 0~15 days, followed by 15~30 days (**Figure 2B**). After 30 days, the growth rate of REC slowed down and tended to be stable after 60 days. The results of MDA content and REC indicated that the cell membrane lipid peroxidation was strong and the cell damage was serious in the first 15 days of storage.

Enzymatic browning is a process in which phenolic substances in plants are oxidized by oxidase into brown quinones, which was reported to be the direct cause of browning in plant tissues such as walnuts (Mittler et al., 2004). Polyphenol oxidase is considered to be the main enzyme involved in plant enzymatic browning (Qian et al., 2021; Fan et al., 2021). In the present study, at 0–15 days of storage, the total phenolic content of walnut endocarps decreased rapidly (from 183.49 to 139.55 mg·g^−1^). It increased at 15-30 days (161.26 mg·g^−1^ at 30 days) and began to decrease at 45 days. The total phenol content increased slowly at 45-75 days and decreased rapidly at 90 days (130.34 mg·g^−1^) (**Figure 2C**). During the whole storage process, PPO activity showed a trend of increasing first and then decreasing. Within 30 days before storage, PPO activity showed an upward trend, with the largest increase within 0-15 days (from 230.91 to 290.27 U g^−1^ min^−1^), and the PPO activity was the strongest at 30 days of storage (314.81 U g^−1^ min^−1^). After 30 days, the PPO activity decreased rapidly with 221.45 U g^−1^ min^−1^ at the end of storage (90 d) (**Figure 2D**).

SOD, CAT, and POD are three important enzymes in the plant antioxidant enzyme system (Ighodaro and Akinloye, 2018). During the storage process (**Figure 2E**), the SOD activity increased rapidly within 0~15 days of storage (from 324.95 to 552.14 U g^−1^) and decreased at 30 days of storage. The SOD activity did not change much during the subsequent storage period, and SOD remained at a high level throughout the storage period (524.64~588.51 U g^−1^). The CAT activity increased during the first 60 days of storage (from 48.61 to 245.90 U g^−1^ min^−1^), decreased rapidly during 60–75 days of storage (102.33 U g^−1^ min ^−1^), and then increased rapidly. After 90 days of storage, the CAT activity was 365.21 U g^−1^ min^−1^. The POD activity mainly decreased during the whole storage period, increased slightly within 45~60 days, and then began to decrease. After the end of storage, the POD activity decreased from 426.40 at 0 days to 202.05 U g^−1^ min^−1^ at 90 days.

The LOX activity showed an alternating pattern of increase or decrease with the rise of storage time. Within 0-15 days, the LOX activity increased significantly, and the first peak (2.02 U g^−1^ min^−1^) appeared and then decreased rapidly to 1.26 U g^−1^ min^−1^ at 45 days (**Figure 2F**). After 60 days, it began to rise again and reached the second peak (2.63 U g^−1^ min^−1^) at 75 days, indicating that the membrane lipid peroxidation was serious at the initial stage of storage. The Na^+^ K^+^-ATPase activity increased significantly within 0~30 days of storage, reaching the first peak (2.24 U g^−1^ h^−1^) at 30 days, and then it reached the second peak (1.98 U g^−1^ h^−1^) at 75 days. Finally, the Na^+^ K^+^-ATPase activity decreased to 0.69 U g^−1^ h^−1^ at 90 days (**Figure 2G**).

To analyze the correlation between the physiological indexes related to the browning of walnut endocarps during storage, the Pearson correlation analysis method was used to analyze the correlation between 10 indexes of walnut during storage at 0~90 days (**Figure 2H**). There were different degrees of correlation between each index. The browning degree was positively correlated with relative conductivity and SOD activity (P< 0.01) and positively correlated with MDA content and LOX activity (P< 0.05). The browning degree was significantly negatively correlated with total phenol content and POD activity (P< 0.05). The browning degree was positively correlated with PPO activity, CAT activity, and Na^+^, K^+^-ATPase activity, but the correlation was not significant (P > 0.05).

### Transcriptome analysis of walnut endocarps of the three different storage periods

To explore the molecular mechanism of walnut endocarps browning during storage, the walnut endocarps at 0, 15, and 30 days with significant differences in nucleolus color were selected for further analysis. 0 days was used as CK (non-browning group), and 15 and 30 days were S15d and S30d (browning group), respectively. Each stage was repeated three times for transcriptome analysis. After removing the low-quality data, the RNA-seq obtained 66.89 Gb clean data from nine samples, the clean data of each sample reached 7 Gb, and the percentage of Q30 bases was 94% or more, indicating that the sequencing data was reliable (**Supplementary Figure S1**; **Supplementary Table S1**).

**Figure 3:**
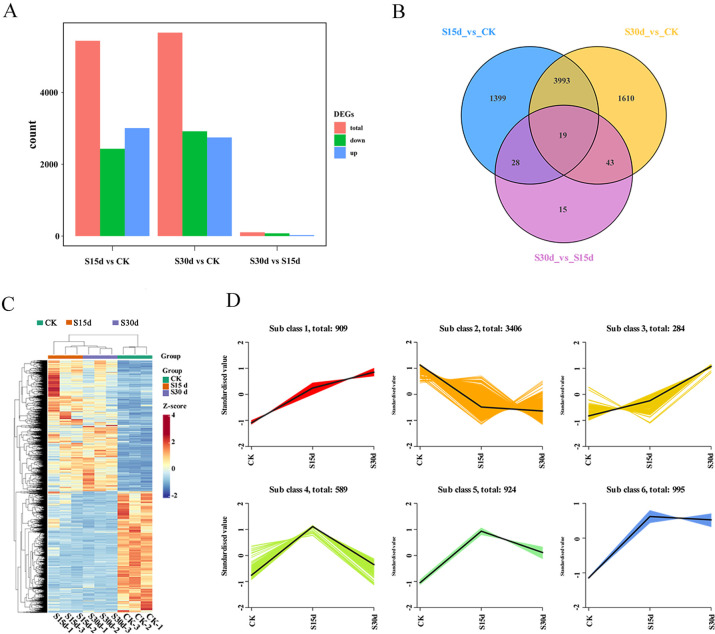
Transcriptional expression of walnut endocarps. **(A)** Number of differentially expressed genes (DEGs) among the three samples. **(B)** Venn diagram of the DEGs. **(C)** Heat map of DEGs based on hierarchical clustering analysis. **(D)** K-means cluster analysis.

The results of principal component analysis (PCA) showed that the first principal component explains 39.48% of the total variance, and the second principal component explains 16.92% of the total variance. The repeatability within the group was good, and CK was significantly separated from S15d and S30d (**Figure 3A**). DEGs were screened between the three groups (|log2Fold Change| ≥1, and P< 0.05). A total of 1,399, 1,610, and 15 DEGs were unique to S15d vs CK, S30d vs CK, and S30d vs S15d, respectively, with 19 DEGs shared by the three comparison groups (**Figure 3B**). The hierarchical clustering results of DEGs in the three comparison groups are shown in **Figure 3C**. Additionally, a total of 5,439 DEGs in S15d vs CK were identified, in which 3,008 genes were upregulated and 2,431 genes were downregulated (**Supplementary Figure S2A**). In the comparison group of S30d vs CK, a total of 5,665 genes were differentially expressed; among them, 2,747 genes were upregulated and 2,918 genes were downregulated (**Supplementary Figure S2B**); In S30d vs S15d, a total of 105 genes showed a differential expression, in which 28 genes were upregulated and 77 genes were downregulated (**Supplementary Figure S2C**).

The results of K-means clustering analysis of DEGs showed that the trend of genes in subclass 1 was consistent with that of endocarps browning. This group of genes mainly included NADH dehydrogenases, cytochrome biosynthesis proteins, photosystem proteins, ATP synthases, ATPases, heat shock proteins, and ribosomal proteins (**Figure 3D**; **Supplementary Table S2**).

**Figure 4:**
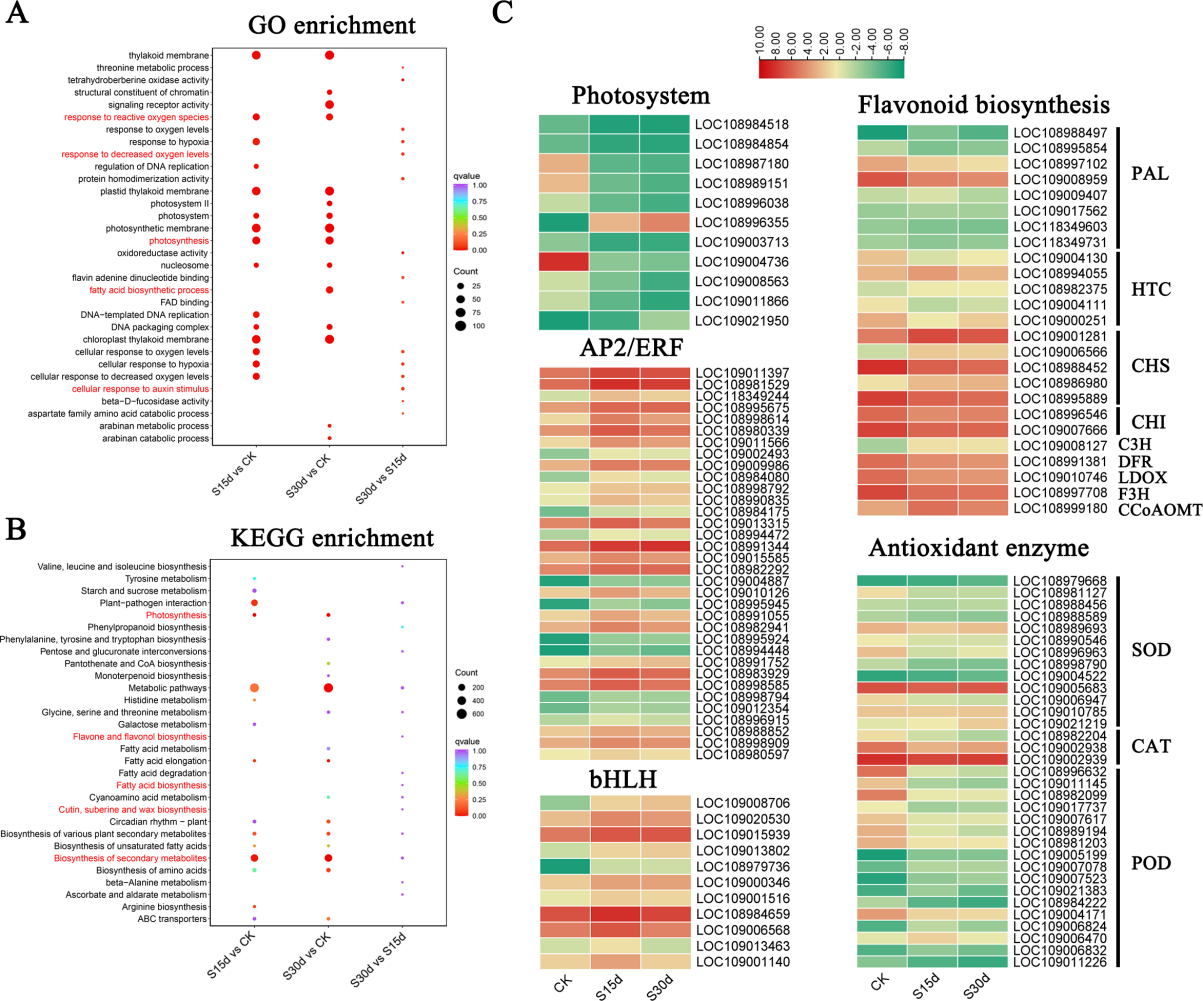
Identification of DEGs in walnut endocarps. **(A)** GO enrichment analyses of DEGs in S15d vs CK, S30d vs CK, and S30d vs S15d. **(B)** KEGG enrichment analyses of DEGs in S15d vs CK, S30d vs CK, andS30d vs S15d. **(C)** Heat map of DEGs involved in flavonoid biosynthesis, antioxidant enzyme genes, photosystem, and transcription factors (AP2/ERF and bHLH), respectively.

The KEGG enrichment analysis showed that DEGs were mainly enriched in biosynthesis of secondary metabolites, plant–pathogen interaction, fatty acid metabolism, biosynthesis of amino acids, saccharo metabolism, and flavonoid biosynthesis of metabolic pathways (**Figures 4A–C**; **Supplementary Figure S4**, **Supplementary Tables S3**, **S4**). Among them, the genes PLA, HTC, CHS, CHI, C3H, DFR, LDOX, F3H, and CCoAOMT related to flavonoid metabolism and antioxidant genes SOD, CAT, and POD were differentially expressed at different storage times (**Figure 4C**; **Supplementary Table S5**). In addition, compared with CK, the expression of a large number of stress-related transcription factors AP2/ERF-ERF, WRKY, C2H2, GARP-G2, bHLH, and HSF and the regulatory factor MYB involved in the secondary metabolic pathway of phenylpropanoids were significantly upregulated at 15 and 30 days of storage (**Figure 4C**; **Supplementary Figure S3**, **Supplementary Table S5**).

### Metabolome analysis of walnut endocarps of the three different storage periods

**Figure 5:**
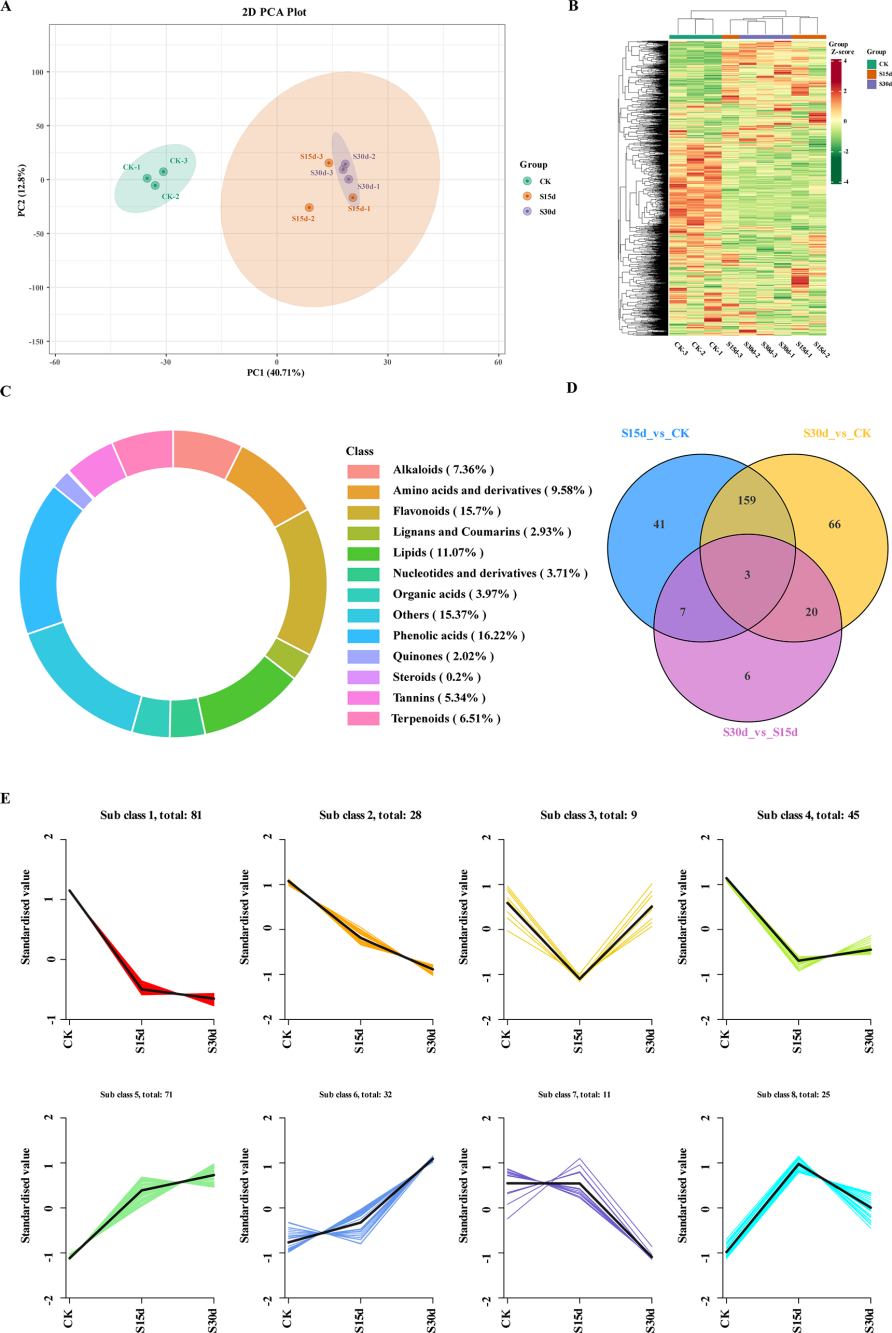
Metabolomics analysis and changes in DAM expression. **(A)** Principal component analysis (PCA). **(B)** Heat map of DAMs based on hierarchical clustering analysis. **(C)** Statistics of metabolites in walnut endocarps. **(D)** Venn diagram of the comparison groups. **(E)** K-means cluster analysis of metabolite expression.

The differential metabolites were analyzed by widely targeted metabolomics. A total of 13 categories of 1,535 metabolites were identified in all the walnut endocarps (**Figure 5C**; **Supplementary Table S4**), in which phenolic acids (16.22%), flavonoids (15.7%), and lipids (11.07%) were the most abundant. Among them, there were 241 kinds of flavonoids, including flavonols (71), flavonoids (45), dihydroflavones (35), other flavonoids (24), flavanols (21), chalcones (19), isoflavones (10), dihydroflavones (8), anthocyanins (2), aurones (2), and dihydroisoflavones (1). The results of the cluster heat map and PCA showed that there were differences in metabolites in walnut endocarps at different storage time (**Figures 5A, B**). In the PCA, component 1 explained 40.71% of the total variance, and component 2 explained 12.8% of the total variance and the repeatability in the group was good. Furthermore, the control group and the browning group were obviously separated, but the separation between S15d and S30d was not obvious, which was consistent with the transcriptome results. The differentially accumulated metabolites (DAMs) were determined by combining the VIP fold change value (VIP > 1, fold change ≥ 2, and fold change ≤ 0.5), and a total of 302 DAMs were detected (**Figure 5D**; **Supplementary Figure S5**). Three identical DAMs were obtained between the three comparison groups (S15d vs CK, S30d vs CK, S30d vs CK S15d), which were flavonoids (2,4,2′,5′-tetrahydroxydihydrochalcone, epigallocatechin-3-O-gallate) and organic acids (phosphoenolpyruvate). A total of 210 DAMs were detected in S15d vs CK, mainly including lipids (43), organic acids (26), amino acids and their derivatives (21), phenolic acids (16), flavonoids (16), and alkaloids (13). A total of 248 DAMs were detected in the S30d vs CK comparison group, including lipids (48), organic acids (26), amino acids and their derivatives (24), phenolic acids (24), flavonoids (22), and alkaloids (10). A total of 36 DAMs were detected in the comparison group of S30d vs S15d, including alkaloids (9), organic acids (4), phenolic acids (4), and flavonoids (4) (**Supplementary Table S4**).

To study the changes of metabolites in different groups, K-means clustering analysis was performed on the metabolites in each comparison group (**Figure 5E**). The results showed that the changes of metabolites in subclass 5 were consistent with the browning degree, mainly including free fatty acids, flavonoids, terpenoids, phenolic acids, and organic acids. The changes of metabolites in subclasses 1 and 2 were opposite to that of the browning degree, mainly including phenolic acids, sugars, amino acids and their derivatives, flavonoids, and organic acids (**Supplementary Table S6**).

The volcanic map was used to determine the upregulation or downregulation of DAMs in each comparison group, and 20 DAMs with the largest difference in refractive index were screened by fold change (**Supplementary Figure S6**). The results showed that compared with CK, 89 metabolites were upregulated (mainly including free fatty acids, flavonoids, and terpenes) and 121 metabolites were downregulated (mainly including amino acids and their derivatives, phenolic acids, flavonoids, and sugars) in the walnut inner seed coat at 15 days of storage (S15d) (**Supplementary Figures S6A, D**; **Supplementary Table S7**). When stored for 30 days (S30d), 104 metabolites were upregulated (mainly including free fatty acids, flavonoids, terpenoids, and phenolic acids) and 144 metabolites were downregulated (mainly including organic acids, phenolic acids, flavonoids, and alkaloids) (**Supplementary Figures SB, E**). Compared with 15 days of storage (S15d), 20 metabolites were upregulated (mainly including sugars, alkaloids, and phenolic acids) and 16 metabolites were downregulated (mainly including alkaloids and flavonoids) at 30 days of storage (S30d) (**Supplementary Figures S6C, F**).

**Figure 6:**
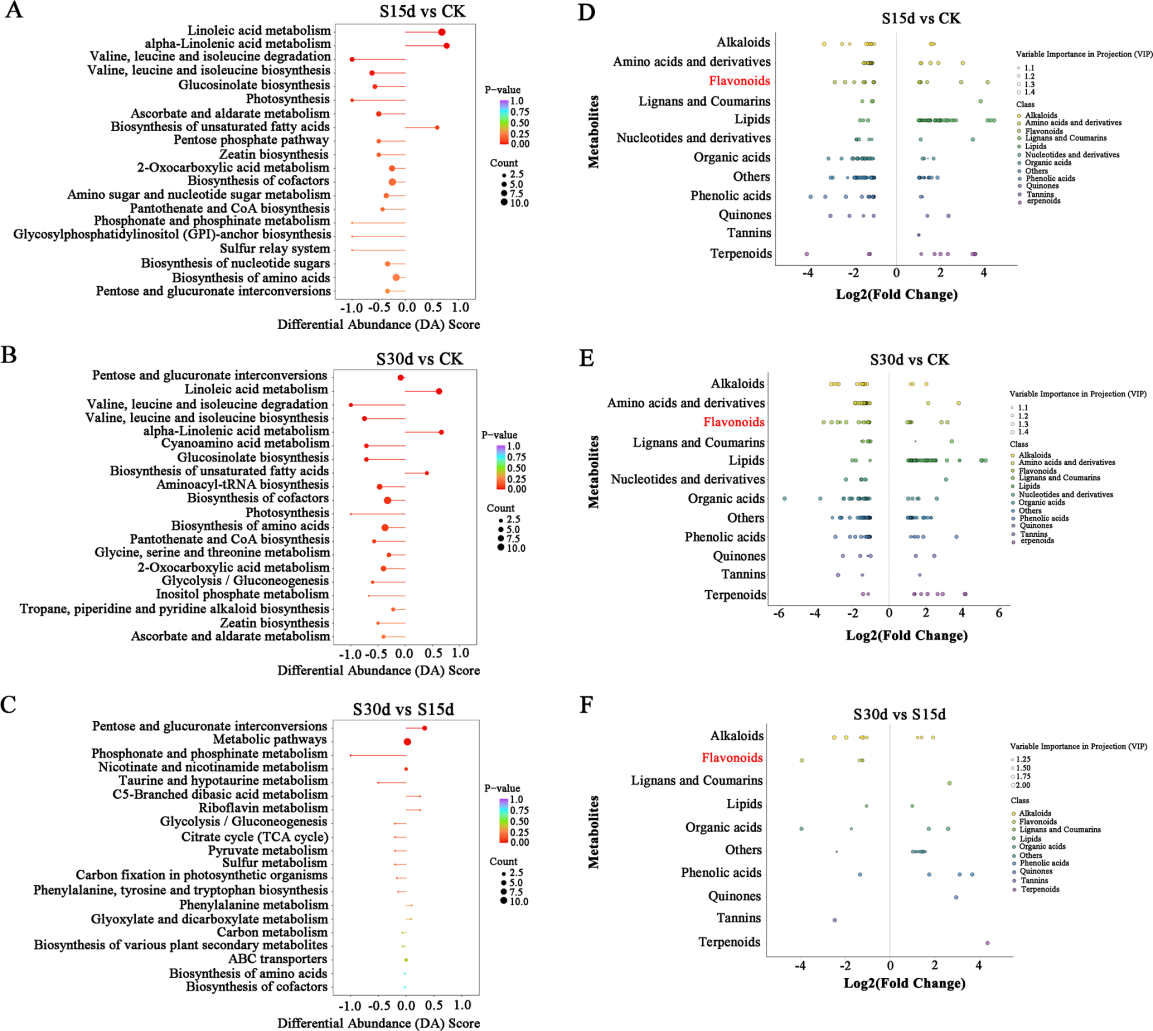
DAM KEGG analysis of each comparison group. DAM selection and KEGG analysis of each comparison group analysis. **(A)** KEGG enrichment analysis of DAMs in S15d vs CK. **(B)** S30d vs CK. **(C)** S30d vs S15d. **(D)** Scatter plot of DAMs in S15d vs CK. **(E)** S30d vs CK. **(F)** S30d vs S15d.

The KEGG enrichment analysis was performed on the DAMs screened in each comparison group to determine the metabolic pathways related to endocarp browning during walnut storage. The top 20 pathways with the highest enrichment in each comparison group were screened by enrichment and topological analysis. The enrichment results of DAM metabolic pathways in each comparison group were basically consistent with the transcriptome results. It was mainly enriched in metabolic pathway, biosynthesis of secondary metabolites, biosynthesis of unsaturated fatty acids, biosynthesis of amino acids, and other metabolic pathways (**Figures 6A–C**). As shown in the scatter plot of differential metabolites (**Figures 6D–F**), metabolites such as lipids, flavonoids, alkaloids, and amino acids and their derivatives were significantly different in different comparison groups (**Supplementary Tables S7**, **S8**).

### Integrated analysis of the transcriptome and metabolome

**Figure 7:**
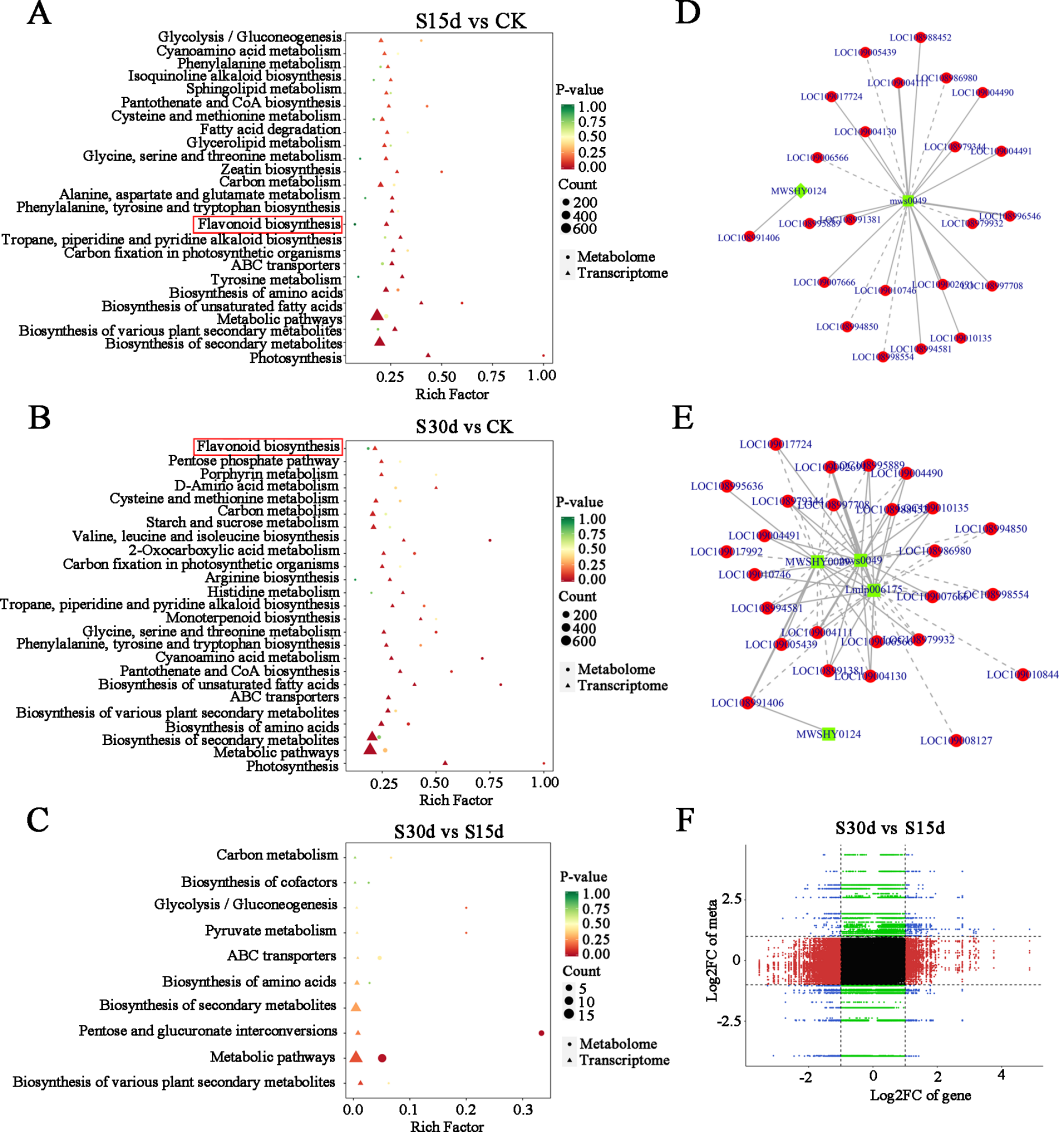
Joint analysis of transcriptome and metabolome. **(A)** KEGG enrichment analysis in S15d vs CK. **(B)** S30d vs CK. **(C)** S30d vs S15d. **(D)** Correlation network of DAMs and DEGs identified from S15d vs CK in flavonoid biosynthesis. **(E)** Correlation network of DAMs and DEGs identified from S30d vs CK in flavonoid biosynthesis. **(F)** Nine quadrants of DAMs and DEGs identified from S30d vs S15d.

In this study, the co-expression network analysis of DEGs and DAMs screened by transcriptome and metabolome was performed (**Figure 7**). The results of KEGG enrichment showed that the main common enrichment pathways of differential genes and metabolites included flavonoid biosynthesis, amino acid biosynthesis, unsaturated fatty acid biosynthesis, phenylalanine metabolism, and oxidative phosphorylation (**Figures 7A–C**). Moreover, the DAMs (pyridinecarboxamide mws0049 and pinocembrin MWSHY0124) were identified to correlate the network with DEGs, which are involved in flavonoid biosynthesis (**Figures 7D, E**).

**Figure 8:**
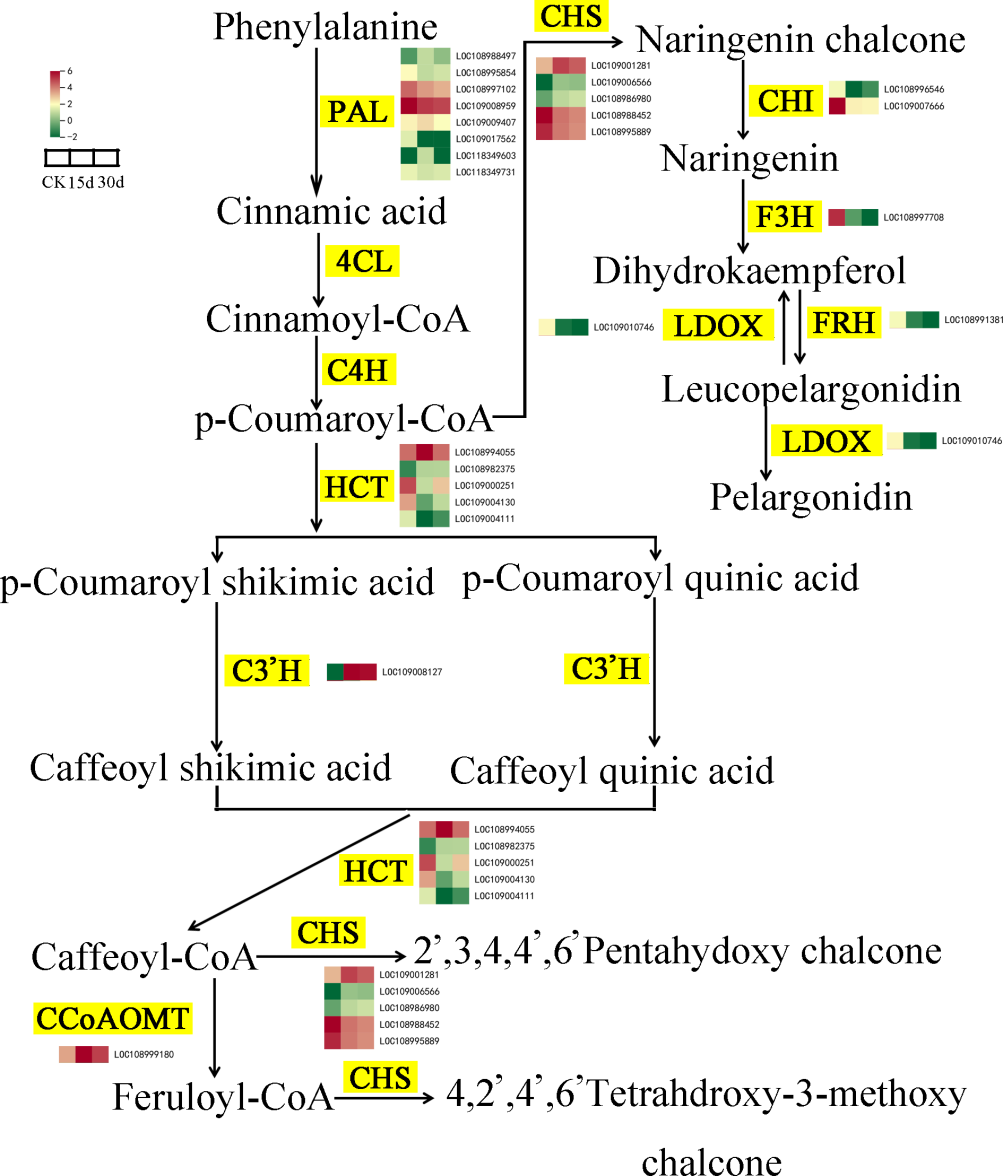
Changes in gene expression and metabolite accumulation levels of the flavonoid biosynthesis pathway. Yellow markings indicate genes, while heatmaps display key transcripts associated with triterpenoid biosynthesis in various tissues.

Phenylalanine is the initial substrate for the synthesis of flavonoids and is catalyzed by phenylalanine ammonia-lyase (PAL), cinnamate 4-hydroxylase (C4H), and 4-coumarate: CoA ligase (4CL) to complete the metabolism of phenylalanine and synthesize p-coumaroyl-CoA. Compared with CK, the expression levels of five PAL genes in the walnut inner seed coat decreased after 15 and 30 days of storage. p-Coumaroyl-CoA is catalyzed by hydroxycinnamoyl transferase (HCT) and chalcone synthase (CHS) to form two flavonol biosynthesis pathways. p-Coumaroyl-CoA, p-coumaroyl shikimic acid, and p-coumaroylquinic acid serve as acyl donors and substrates and are catalyzed by HCT to form caffeoyl shikimic acid and caffeoyl quinic acid, which are then catalyzed again to form caffeoyl-CoA, and finally under the action of CHS, 2′,3,4,4′,6′-pentahydoxychalcone and 4,2′,4′,6′ and tetrahdroxy-3-methoxy chalcone are synthesized. After 15 and 30 days of storage, HCTs showed differential expression (two upregulated, three downregulated). In addition, the molecular weight of p-coumaroyl-CoA and malonyl-CoA is 3:1, which are converted into naringenin through a two-step condensation reaction with CHS and chalcone isomerase (CHI), which is then oxidized by flavanone 3-hydroxylase (F3H) to generate dihydrokaempferol, and finally synthesized into anthocyanidin and pelargonidin under the action of leucoanthocyanidin reductase (LAR). CHS genes in the flavonoid biosynthesis pathway were differentially expressed (three upregulated, two downregulated), and the expression levels of CHI, flavanone 3-hydroxylase (F3H), leucocyanidin dioxygenase (LDOX), and FRH decreased after 15 and 30 days of storage (**Figure 8**).

In addition, the nine-quadrant diagram was drawn by using the absolute value of Pearson correlation coefficient greater than 0.8 and p< 0.05, and the changes of each stage were systematically compared (**Figures 7D–F**). The results showed that there was a significant negative correlation between DEGs and DAMs in quadrants 1 and 9, while there was a significant positive correlation in quadrants 3 and 7. According to the distance to the origin of the O2PLS model, the relationship between these DEGs and DAMs was studied. The results showed that cytochrome synthesis gene (LOC108993766), WRKY transcription factor (LOC108984580), threonine-protein kinase (LOC108991876), xyloglucan and glycosyltransferase (novel.2317, LOC109013464) in the transcription level were significantly correlated with the expression of metabolites (**Supplementary Table S9**). In the metabolic group, the phenolic acids (4-hydroxybenzoic acid propyl ester MWS2070), flavonoids (eriodictyol-7-O-(6''-malonyl) glucoside zbnn005420), organic acids (trans-aconitic acid pme3009, 4-hydroxy-2-ketoglutarate pme2761, azelaic acid mws0237), quinones (4,8-dihydroxy-1-tetralone wmhn005790), alcohols (2,6-dimethyl-7-octen-2,3,6-triol pmf0348), aldehydes (4-hydroxyphenylacrolein mwscx017), lactone (senkyunolide mwsmce412), and other substances were significantly correlated with DEGs (**Supplementary Figure S6B**; **Supplementary Table S10**).

## Discussion

Walnuts were prone to browning during postharvest storage, which affects the taste and flavor of kernels and reduces their commercial value. Therefore, the browning has become a major factor affecting the storage of walnut nuts and limiting the shelf life of kernel products (Villarreal-Lozoya et al., 2009). In this study, combined with the results of endocarp color and browning-related indicators, it was not difficult to find that the browning of walnuts was the most serious in the first 15 days of accelerated storage. After 60 days, the browning rate changed slowly, and the browning degree gradually increased throughout the storage period, which was consistent with the research results of Asaad et al. (Habibie et al., 2019). This may be the high water content of nucleolus and the high activity of oxidation-related enzymes in the early stage of storage.

The content of related substances in phenolic metabolism and the activity of related enzymes significantly affect the occurrence of post-harvest browning of fruits (Cosmulescu et al., 2014). Walnut endocarps are rich in phenolic substances. On the one hand, phenolic substances have strong antioxidant activity, which can protect the nucleolus from the oxidation of fatty acids and protect the quality of nucleolus. On the other hand, phenolic substances will be used as substrates for enzymatic browning, which will be oxidized under the catalysis of PPO and cause browning (Burdon et al., 2007). In this study, with the increase of browning degree, the total phenolic content showed a downward trend, which was consistent with the fact that the expression of phenolic acids was significantly downregulated at 15 and 30 days of storage compared with CK in metabolomics analysis. In addition, the PPO activity increased significantly in the early stage of storage, indicating that the enzymatic browning of polyphenol oxidation catalyzed by PPO in the first 30 days of storage was the main reason for the browning of walnut inner seed coat and then decreased rapidly after 45 days, which may be the inhibition of PPO activity with the accumulation of enzymatic browning products (Ho et al., 2013).

SOD, CAT, and POD were very important enzymes in the plant tissue membrane protection system, which are closely related to tissue browning (Ighodaro and Akinloye, 2018; Zhang et al., 2025). Among them, SOD as the first barrier of the antioxidant system will first scavenge free radicals and produce H_2_O_2_ accumulation and then scavenge H_2_O_2_ by enzymes such as CAT and APX, thereby reducing the accumulation of reactive oxygen species (Zhu et al., 2016). In this study, SOD activity increased rapidly in the early stage of storage (15 days), but CAT activity increased slowly, indicating that SOD was the main enzyme to remove reactive oxygen species in the early stage of storage. With the extension of storage time, the activity of SOD increased slowly, and even decreased, while the activity of CAT increased significantly, which may be the serious aging and damage of cells with the extension of storage time, which affected the synthesis of SOD-related proteins (Yan et al., 2002). At the same time, a large amount of H_2_O_2_ was produced, which enhanced the activity of CAT. POD was widely present in plant tissues and had strong activity. It was a heme-containing oxidoreductase that can scavenge H_2_O_2_ produced by cell peroxidation and has an important impact on plant growth and development and stress resistance defense. In this study, POD activity showed a downward trend within 0~45 days of storage and increased after 45 days (**Figures 2**, **4**). It may be the fact that POD was mainly used as an oxidase in the early stage of storage and participated in the oxidation function of catalyzing phenols together with PPO. Since POD can catalyze the oxidation of phenolic substances in the presence of H_2_O_2_, POD-related genes cause tissue browning (Aquino-Bolaños and Mercado-Silva, 2004). With the extension of storage time, the total phenol content of the oxidation substrate decreased, resulting in a decrease in POD activity. Later, with the deepening of membrane lipid peroxidation, more free radicals and H_2_O_2_ were produced, and POD was involved in the removal of H_2_O_2_, resulting in an increase in its activity. Some other studies showed that the relationship between POD activity and browning is related to plant varieties (Franck et al., 2007). Therefore, the relationship between POD and walnut browning remains to be further studied.

The cellular membrane system has the great function of maintaining cell homeostasis and ensuring normal metabolism. The cell biofilm structure in the fruit is generally in a dynamic equilibrium state. Once the balance is broken, the cell structure will be destroyed, which will lead to browning (Scotti-Campos et al., 2014). Some studies showed that the membrane metabolism disorders cause damage to the membrane structure, which promotes the contact reaction between the substrate and the enzyme to accelerate the browning phenotype (Mayer and Harel, 1979). It was found that the membrane lipid metabolism of longan fruit was affected after infection with mold, and the activities of some enzymes related to membrane lipid degradation, such as PLD and LOX, were abnormally increased, and the fruit showed a browning phenotype (Wang et al., 2018). In this study, LOX activity, MDA content, and relative conductivity showed an upward trend with the prolongation of storage time, and LOX activity and MDA content were significantly positively correlated with browning degree (P< 0.05), and relative conductivity was significantly positively correlated with browning degree (P< 0.01). At the same time, the results of transcriptomics analysis showed that DEGs in different storage periods were enriched in reactive oxygen species metabolism, photosynthetic membrane, chloroplast thylakoid membrane, plastid thylakoid membrane, thylakoid membrane, and other functions. The results of metabolomics analysis also showed that compared with CK, the cumulative expression of a large number of free fatty acids was upregulated at 15 and 30 days of storage. In addition, the cell ultrastructure also showed that from 15 days of storage, the cell structure began to change, the vacuole had inclusion exudation, the mitochondria began to disintegrate far away from the cell wall, and the electron density of the cell wall began to decrease. After 30 days, the cell wall began to thicken and the inclusions were severely degraded. After 60 days of storage, the cell wall began to crack. These results indicated that lipid oxidation and differential expression of related genes are one of the important reasons for browning of walnut inner skin during storage.

Energy is the basis of fruit postharvest metabolism, which is of great significance to maintain normal life metabolism after fruit harvest (Li et al., 2019). Energy supply plays an important role in controlling fruit senescence and postharvest physiological disorders. As the key enzyme in energy metabolism and regulation of ATP synthesis, ATPase plays an important role in the regulation of fruit browning. The decrease of its activity will cause cell energy loss and browning (Liu et al., 2006). Some scholars believe that energy metabolism plays an important role in maintaining the structure and function of cell membranes (Lin et al., 2016). For example, energy loss can lead to cell membrane repair dysfunction (Rawyler et al., 2002). In recent years, more and more studies showed that intracellular energy is essential for the synthesis of phospholipids and the repair of cell membranes (Wang et al., 2020). ClO2 treatment of longan fruits could enhance the activity of enzymes related to energy metabolism, such as NADH SDH, CCO, SCS, and ATPase, to promote and maintain energy production, thereby improving the overall energy level and reducing browning (Vichaiya et al., 2020). In addition, free radicals have a certain damage effect on Na^+^ K^+^-ATPase; when the cell membrane peroxidation is enhanced, the Na^+^ K^+^-ATPase activity will be significantly reduced. The results of this study showed that with the prolongation of storage time, the activity of Na^+^ K^+^-ATPase increased first and then decreased. It may be the damage of cell structure caused by membrane lipid peroxidation in the early stage. The body starts the repair mechanism and needs ATP to supply energy. Therefore, the activity of ATPase increases accordingly. With the prolongation of storage time, the accumulation of free radicals produced by enhanced oxidation causes damage to ATPase and leads to a decrease in its activity.

Transcriptome analysis showed that compared with CK, the expression of transcription factors involved in environmental stress was significantly upregulated at 15 and 30 days of storage, such as AP2/ERF-ERF, WRKY, C2H2, GARP-G2, bHLH, and HSF, indicating that high-temperature stress, senescence, and cell dehydration during storage of walnuts are important factors causing browning (Yang et al., 2024). Previous study has reported that WRKYs can promote the transcriptional reprogramming during banana peel browning (Zhu et al., 2023). At the same time, the expression of cell wall structural protein gene was also significantly upregulated, which was consistent with the results of cell wall thickening at 30 days of storage in cell ultrastructure observation. In addition, the regulatory factors (MYB) involved in phenylpropanoid metabolism and antioxidant system glutathione transferase genes (GSTF, GSTU) were also significantly expressed, which indicated that the browning of walnut endocarps during storage was related to the metabolism of this propane (Zhang et al., 2022). MYB–bHLH–WDR protein complexes have been reported to have transcriptional control of flavonoid biosynthesis (Xu et al., 2015). In our study, the differential expression of MYB and bHLH genes (**Figure 4**; **Supplementary Figure S3**) suggested that they are involved in the flavonoid biosynthesis. Furthermore, the downregulation of PAL and CHI genes correlates with reduced total phenolic content, which associated with epigallocatechin-3-O-gallate in the flavonoid biosynthetic pathway (**Figure 4**; **Supplementary Table S4**), indicating that PAL, CHI, and POD genes were involved walnut endocarp browning. Phenylpropanoids were a class of compounds including tannins, anthocyanins, melanin, and lignin, and many of these compounds are colored and play a role in coloring and antioxidant protection for plants (Grace and Logan, 2000; Korkina, 2007).

Flavonoids can inhibit the production of ROS and play an important role in the response of plants to abiotic stress (Yan et al., 2002). The results of metabolomics analysis in this study showed that a total of 241 flavonoids were detected in the walnut endocarps during storage, accounting for a large proportion of the metabolites, and the analysis of differential metabolites showed that compared with CK, the contents of phenols, amino acids and their derivatives, sugars, and other substances decreased, and the contents of free fatty acids, flavonoids, quinones, and other substances increased at 15 and 30 days of storage. At the same time, the results of transcriptome and metabolome combined analysis also showed that differential genes and metabolites were mainly enriched in flavonoid biosynthesis, amino acid biosynthesis, unsaturated fatty acid biosynthesis, phenylalanine metabolism, and oxidative phosphorylation.

## Data Availability

The datasets presented in this study can be found in online repositories. The names of the repository/repositories and accession number(s) can be found in the article/**Supplementary Material**.
