# Supplementary material for: Integrated metabolomic and transcriptomic analysis provides insights into the browning of walnut endocarps
Source: Front Plant Sci. 2025 May 9;16:1582209. doi: 10.3389/fpls.2025.1582209 (PMC12098456; doi:10.3389/fpls.2025.1582209)
Supplement: Supplementary file 1 [file Image1.pdf]

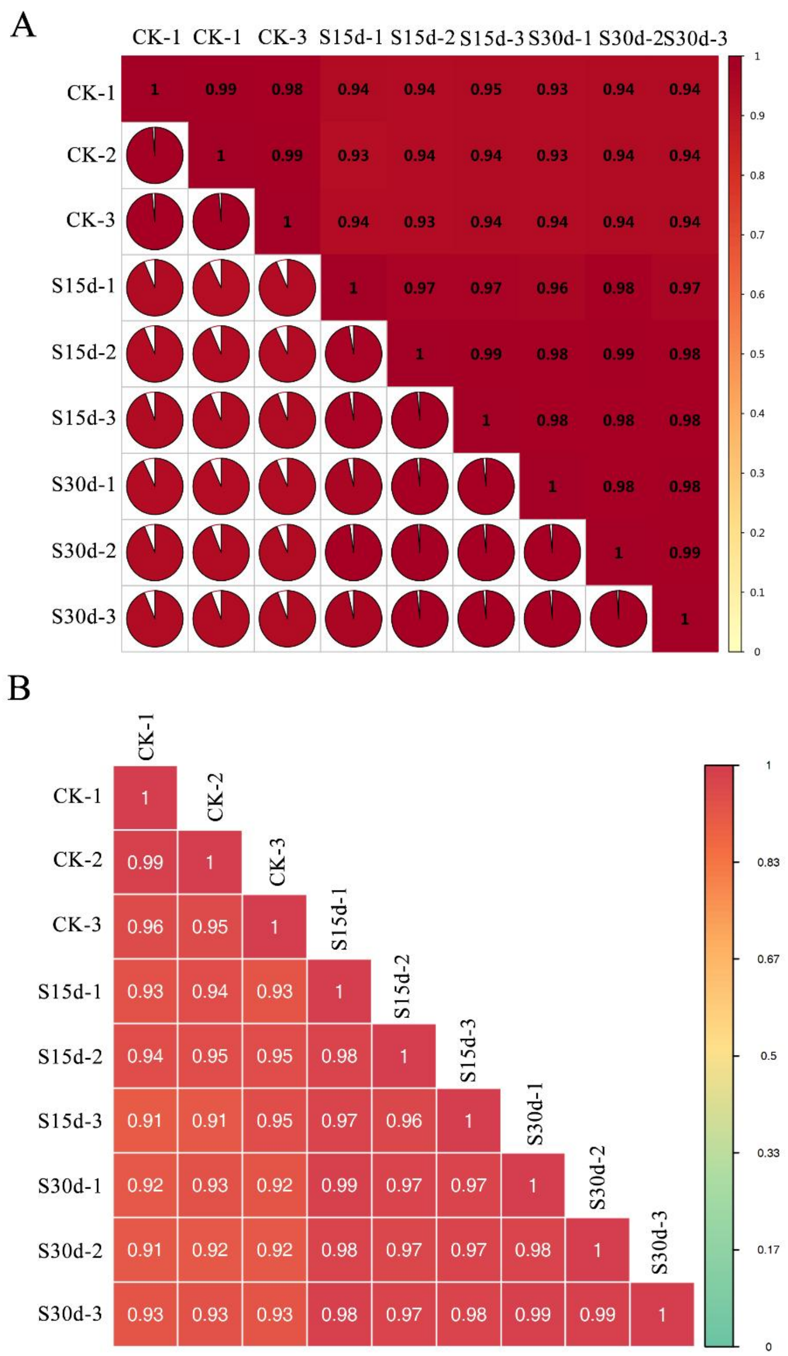

Figure S1: The correlation relationship among different samples for transcriptome and metabolome analysis.

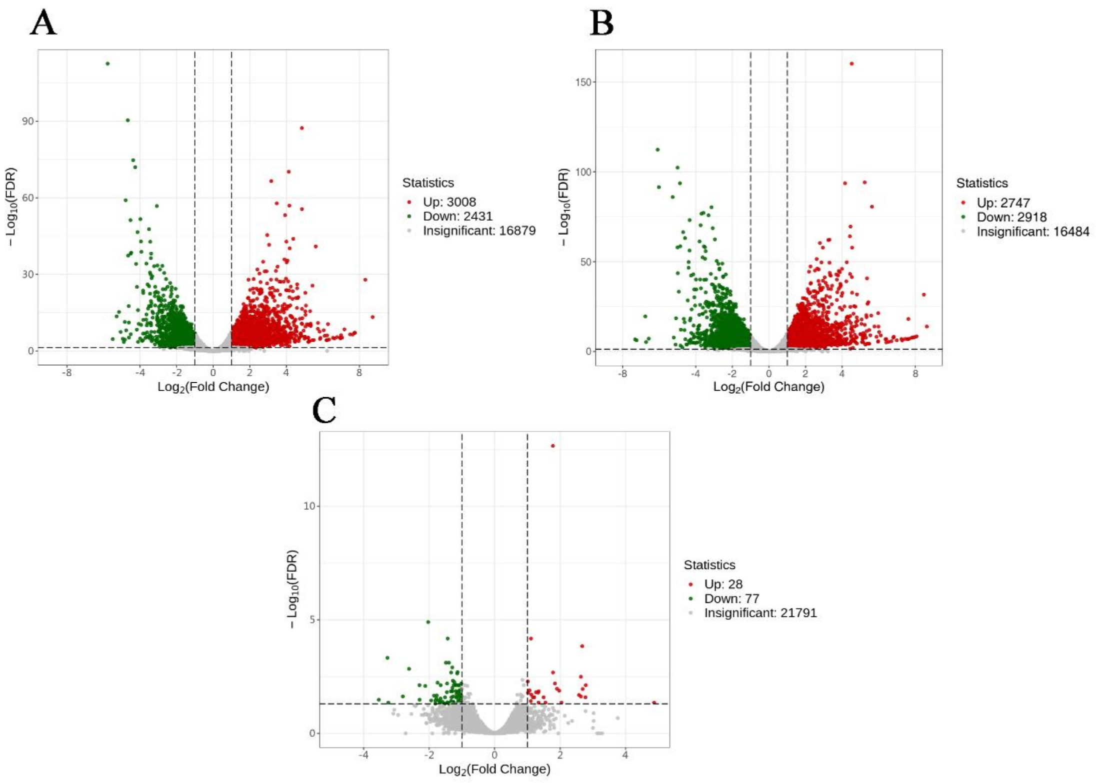

Figure S2: The volcano map analysis of differential expressed genes (DEGs) during the storage period.

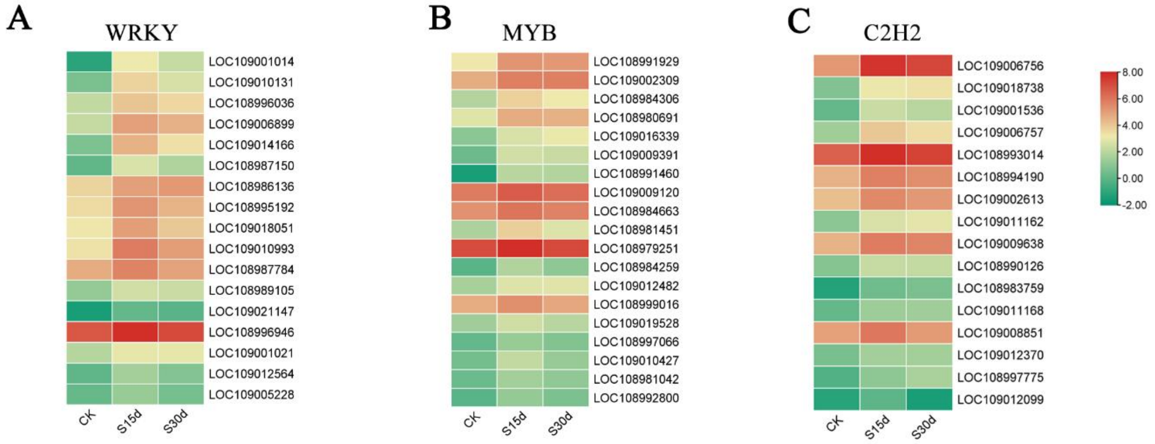

Figure S3: The differential expressed genes (DEGs) of three transcription factors (WRKY, MYB and C2H2) during the storage period.

A

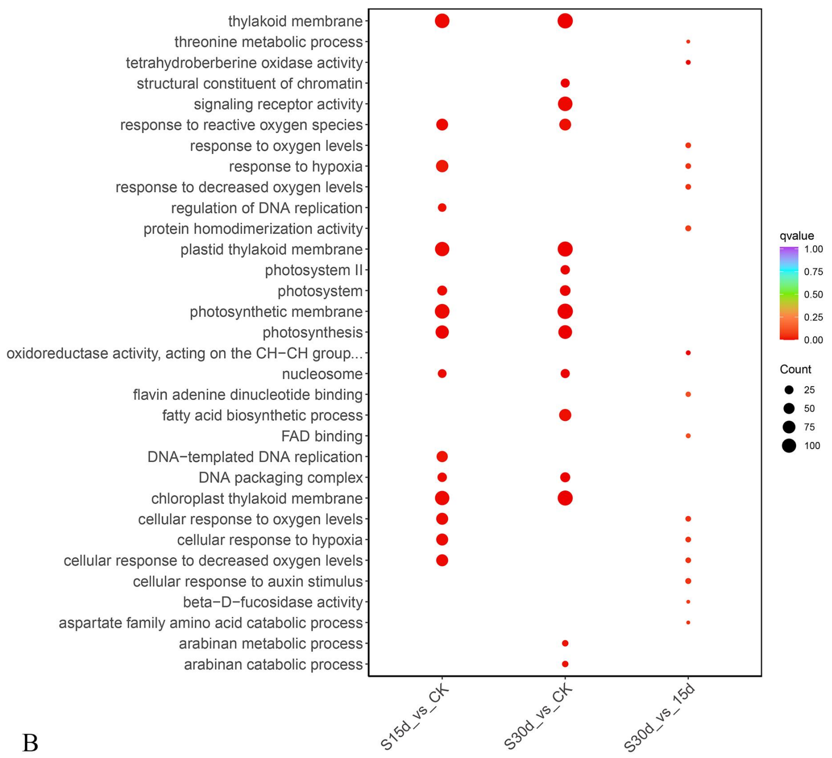

B

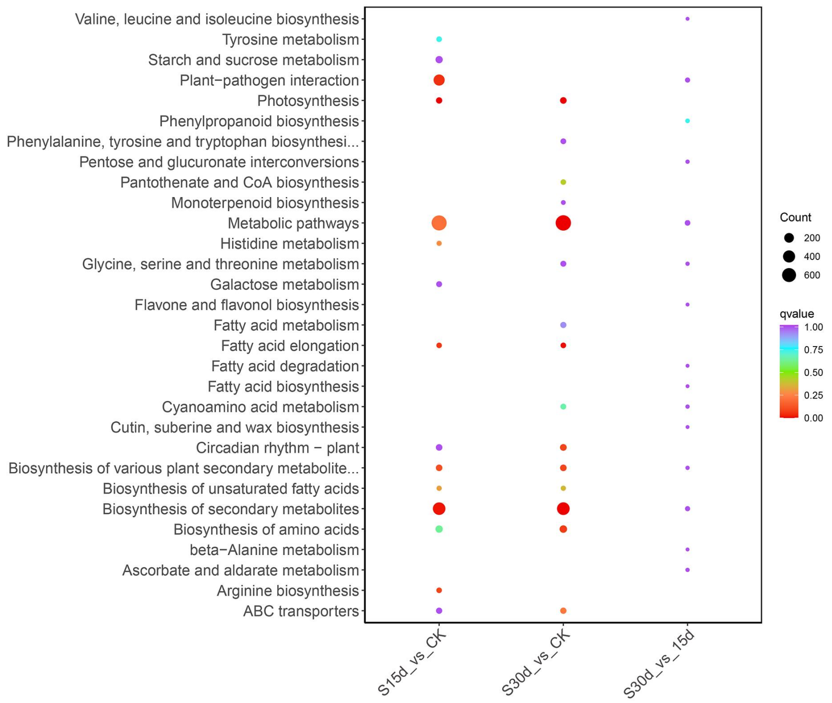

Figure S4 GO and KEGG enrichment analysis of the three comparisons during the storage period. (A) GO enrichment; (B) KEGG enrichment. The red boxs indicate the key biological process and pathway, respectively.

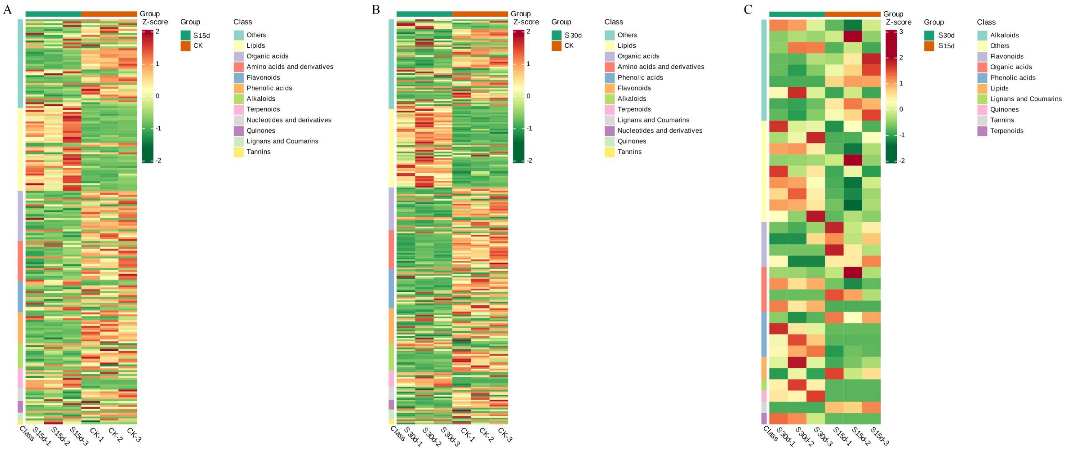

Figure S5 The heatmaps of the all metabolites in the three comparisons during the storage period. (A) S15d vs CK; (B) S30d vs CK; (C) S30d vs S15d.

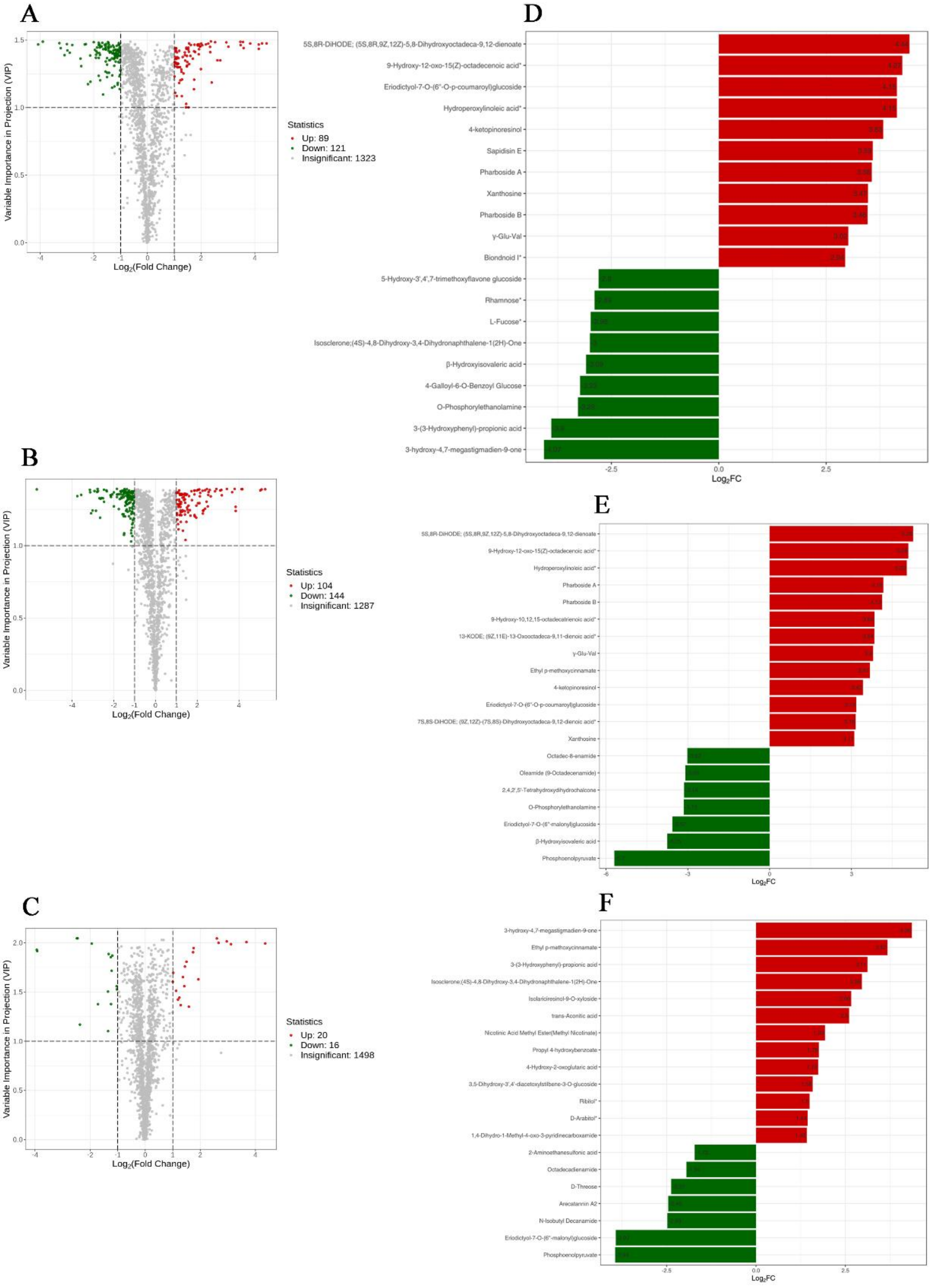

Figure S6 The volcano map and bar chart of differential metabolites during the storage period.
